# Vitamin D and the risk of treatment-resistant and atypical depression: A Mendelian randomization study

**DOI:** 10.1038/s41398-021-01674-3

**Published:** 2021-11-04

**Authors:** Ryan Arathimos, Amy Ronaldson, Laurence J. Howe, Chiara Fabbri, Saskia Hagenaars, Matthew Hotopf, Fiona Gaughran, Cathryn M. Lewis, Alexandru Dregan

**Affiliations:** 1 Social, Genetic and Developmental Psychiatry Centre, Institute of Psychiatry, Psychology and Neuroscience, King’s College London, London, UK; 2 NIHR Maudsley Biomedical Research Centre, South London and Maudsley NHS Trust, London, UK; 3 Department of Psychological Medicine, Institute of Psychiatry, Psychology and Neuroscience, King’s College London, London, UK; 4 MRC Integrative Epidemiology Unit, Bristol Medical School, University of Bristol, Bristol, UK; 5 Department of Biomedical and Neuromotor Sciences, University of Bologna, Bologna, Italy; 6 South London and Maudsley NHS Foundation Trust, London, UK; 7 Psychosis Studies Department, Institute of Psychiatry, Psychology and Neuroscience, King’s College London, London, UK; 8 Department of Medical and Molecular Genetics, Faculty of Life Sciences and Medicine, King’s College London, London, UK

**Keywords:** Depression, Clinical genetics

## Abstract

Observational evidence has implicated vitamin D levels as a risk factor in major depressive disorder (MDD). Confounding or reverse causation may be driving these observed associations, with studies using genetics indicating little evidence of an effect. However, genetic studies have relied on broad definitions of depression. The genetic architecture of different depression subtypes may vary since MDD is a highly heterogenous condition, implying potentially diverging requirements in therapeutic approaches. We explored the associations between vitamin D and two subtypes of MDD, for which evidence of a causal link could have the greatest clinical benefits: treatment-resistant depression (TRD) and atypical depression (AD). We used a dual approach, combining observational data with genetic evidence from polygenic risk scores (PRS) and two-sample Mendelian randomization (MR), in the UK Biobank. There was some evidence of a weak association between vitamin D and both incident TRD (Ncases = 830) and AD (Ncases = 2366) in observational analyses, which largely attenuated when adjusting for confounders. Genetic evidence from PRS and two-sample MR, did not support a causal link between vitamin D and either TRD (Ncases = 1891, OR = 1.01 [95%CI 0.78, 1.31]) or AD (Ncases = 2101, OR = 1.04 [95%CI 0.80, 1.36]). Our comprehensive investigations indicated some evidence of an association between vitamin D and TRD/AD observationally, but little evidence of association when using PRS and MR, mirroring findings of genetic studies of vitamin D on broad depression phenotypes. Results do not support further clinical trials of vitamin D in these MDD subtypes but do not rule out that small effects may exist that require larger samples to detect.

## Introduction

Major depressive disorder (MDD) is the leading cause of disability globally affecting 4.7% of the population [1]. Vitamin D deficiency is a major global health problem [2] and in the UK rates of both vitamin D deficiency and insufficiency are considerable [3]. Vitamin D is considered essential for bone health and immune function [4], but is also thought to affect the expression of certain neurotransmitters. Vitamin D receptors are present on several brain areas known to be implicated in depression [5]. Existing observational evidence suggests that vitamin D status might be a risk factor for the development of MDD. Several meta-analyses of longitudinal studies have shown that vitamin D status is prospectively associated with depression in older adults [6–8]. Using a sample three times larger than the world literature (*n* = 139,128), a recent study has shown that both vitamin D deficiency and insufficiency are associated with new onset depression in middle-aged adults [9].

Although observational evidence implies that vitamin D status might play a role in the development of depression, these associations could be subject to unmeasured confounding and so provide limited evidence regarding causality. Results from trials assessing the effects of vitamin D supplementation on depression have been mixed. Some meta-analyses have concluded that vitamin D supplementation has no effect on depressive symptoms [10, 11] whereas the most recent meta-analysis has shown that vitamin D reduces depression ratings in patients with MDD [12]. There is some evidence that vitamin D used as an adjunctive therapy to antidepressants can improve depressive symptomatology in patients with MDD across the lifecourse [13–15].

An alternative way to establish whether there is a causal role for vitamin D in MDD is through the use of genetic information. Polygenic risk scores (PRS) leverage results from large genome-wide association studies (GWAS) and combines information across many genetic loci, capturing part of an individual’s genetic susceptibility to a disease or genetic predisposition to a trait [16]. Previous GWAS have identified a number of genetic variants associated with vitamin D levels [17–19], which can also be used in Mendelian randomization analyses [20] to evaluate the effects of vitamin D on MDD. The premise is that if genetic variants associated with higher vitamin D are also associated with increased risk of MDD, then this indicates a potential causal relationship between vitamin D and MDD. The advantages of a Mendelian randomization approach are that it can largely rule out reverse causation because germline genotype is fixed from conception, and that it is less susceptible to confounding. Several recent Mendelian randomization studies have indicated little evidence of a causal association between vitamin D status and depression [19, 21–24]. However, these studies have relied on broad definitions of depression or depressive symptoms to increase sample size and therefore statistical power, with some indications that genetic architecture of different depression subtypes; particularly those that incorporate a more strictly defined MDD phenotype, could be very different [25].

MDD is a heterogenous mental health condition with several subtypes, implying diverse biological underpinnings and potentially diverging requirements in therapeutic approaches. One such subtype is treatment-resistant depression (TRD). Common treatments for MDD include antidepressant drugs and psychotherapies. Approximately 30% of MDD patients do not respond to antidepressants or psychotherapy and are referred to as having TRD [26]. Vitamin D deficiency may be involved in the persistence of depressive symptoms despite antidepressant treatment, in these MDD patients. Another subtype of MDD is atypical depression (AD). The Diagnostic and Statistical Manual of Mental Disorders 5th edition (DSM-5) defines AD as depression that comprises mood reactivity and at least two of the following symptoms: hypersomnia, increased appetite or weight gain, leaden paralysis, and interpersonal rejection sensitivity [27]. Approximately 15–29% of patients with MDD have AD [28], which is associated with more chronic course of disease and poorer response to treatment [29]. Currently, there is a critical need for new treatments for patients with TRD [30], and treatment guidelines for AD are lacking [31].

In the current study, we use both observational and genetic information to evaluate the effects of exposure to higher vitamin D on the risk of TRD and AD, in order to assess the case for potential trialling of vitamin D supplementation in these patient groups.

## Methods

### Data

#### UK Biobank

The UK Biobank is a prospective cohort study of over 500,000 individuals across the UK. Participants were aged 40–69 years at recruitment in 2006–2010 [32]. In addition to phenotype data which included assays of biomarkers from blood draws at baseline, genotype data were available in all UK Biobank participants [33]. Ethical approval was granted by the NHS North West Research Ethics Committee (REC reference 11/NW/0382). Written informed consent was obtained from all participants. In a follow-up of the full cohort, 157,366 UKB participants completed an online Mental Health Questionnaire (MHQ), designed to assess the presence and severity of mental health conditions, beginning in 2017 [34]. Primary care electronic health records (EHR) were available for approximately 45% of the cohort (~230,000 participants), all of whom have provided written consent for linkage to their health-related records, containing diagnostic codes and prescription drug history, with the majority of records beginning in 1985, up until 2017.

### Phenotype data

#### Vitamin D

Serum levels of 25-hydroxyvitamin D (25OHD, nmol/L) were measured using a chemiluminescent immunoassay (DiaSorin Liaison XL, DiaSorin Ltd., UK) from blood draws at baseline (2006–2010). The assay for 25OHD had an analytical sensitivity (lower detection limit) of 10 nmol/L. The coefficient of variation ranged from 5.04 to 6.14%. Values greater that 3x the interquartile range (IQR) from the mean were excluded (*N* = 350, 0.07% of total). Measures of vitamin D were then ranked inverse-normal transformed.

#### Treatment-resistant depression

Prevalent TRD was defined using primary care data. Cases were defined as individuals with at least two diagnostic codes for any unipolar depressive disorder (probable MDD) and at least two switches between antidepressant drugs, each prescribed for at least six weeks, as previously described [35]. Incident TRD cases were defined as those whose first antidepressant drug prescription (used to define TRD status) occurred after the blood draw date. Controls were either prevalent probable MDD cases, as defined from primary care data, that did not satisfy criteria for TRD (MDD controls), or participants with primary care data that did not satisfy criteria for TRD (whole sample controls). In a sensitivity analysis, we excluded probable MDD cases, as defined from the EHR data, from the whole sample controls.

#### Atypical depression

Atypical depression (AD) was defined based on self-reported depressive symptoms in the follow-up MHQ. Cases were defined as individuals who met the DSM-5 criteria for probable lifetime MDD based on the Composite International Diagnostic Interview (CIDI) Short Form and who reported both hypersomnia and weight gain, as previously described [29]. In all cases, atypical depression was defined approximately 8–9 years after vitamin D measurement (baseline). Controls were either individuals who met criteria for probable lifetime MDD (MDD controls) based on the MHQ, but did not satisfy criteria for AD, or participants who responded to the MHQ but did not satisfy criteria for AD (whole sample controls). In a sensitivity analysis, we excluded probable MDD cases, as defined from the MHQ, from the whole sample controls.

#### Covariates

All covariates were measured at the baseline assessment and included sex, age, smoking (never, current or past smoker), Townsend deprivation index [36] (TDI—as a proxy for socioeconomic status), body mass index (BMI), ethnicity (white/non-white), and alcohol consumption frequency (daily/almost daily, 1–4 times per week, 1–3 times a month, never), and season of blood draw (summer, winter, spring or autumn) [37].

### Genetic data

Genotypes in UK Biobank were assayed using two different arrays (chips), the Affymetrix UK BiLEVE Axiom or Affymetrix UK Biobank Axiom array. Preliminary quality control (QC) on genetic data was performed internally by UK Biobank and additional QC steps are described in Supplementary Methods.

### Statistical analyses

#### Observational associations

We used logistic regression to test associations between vitamin D and subtypes of depression as the outcome, with stepwise adjustment for covariates, which included age, sex, BMI, TDI, ethnicity, smoking, alcohol consumption frequency, and season of blood draw. We conducted a complete case analysis in the observational analysis for each depression subtype, where individuals with missingness in covariates were removed.

#### Genetic associations

##### Polygenic risk score analysis

PRS for vitamin D were calculated from summary statistics from the largest published genome-wide association study of vitamin D (that did not include UK Biobank), conducted in a sample of 79,366 European-ancestry individuals by the SUNLIGHT consortium [18]. PRS was calculated using PRSice v2 [38, 39], with clumping at an r2 < 0.1 and a 500 kb window. PRS were calculated at 11 *p*-value thresholds and the PRS threshold with the highest predictive power (when compared against serum vitamin D in the UK Biobank) based on the R2 value once the first six genetic PCs were taken into account, was used in downstream analyses. We tested for an association between the most predictive PRS and the depression subtypes using logistic regression in R 3.6.0, adjusting for the first six genetic PCs. In a sensitivity analysis, we adjusted additionally for the assessment centre and genotype batch. We also performed a power calculation for the PRS analyses using the R package avengeme [40, 41].

##### Mendelian randomization

Mendelian randomization (MR) is a type of instrumental variable (IV) analysis that can strengthen causal inference by using genetic variants as proxies for exposures [20, 42]. Two-sample MR is an extension to the method where the effects of the genetic IVs on the exposure and on the outcome are calculated in separate datasets or obtained from separate GWAS. MR makes three key assumptions: firstly, the genetic variants must be robustly associated with the exposure of interest, secondly, genetic variants must not be associated with potential confounders of the exposure and outcome, and thirdly, there must be no effects of the genetic variants on the outcome, that are not via the exposure (horizontal pleiotropy).

Two-sample Mendelian randomization was performed using the TwoSampleMR package [43]. We used two different but complementary sets of IVs in the MR. The first (IV set A) was derived from a GWAS of vitamin D in the subsample of UK Biobank without MHQ/primary care data, and the second (IV set B) from a previously conducted whole-sample GWAS of vitamin D in UK Biobank [19]. For IV set A (*N* = 149,607), GWAS were conducted using regenie [44], a mixed model method based on ridge regression, with correction for the first six genetic PCs. We selected independent genome-wide significant variants by clumping, using the European subset of 1000 Genomes panel [45] as the reference (details of the GWAS in Supplementary Methods). IV set B SNPs were extracted from the whole-sample GWAS of vitamin D conducted in UK Biobank (*N* = 417,580) [19]. We clumped GWAS summary results using the European subset of 1000 Genomes panel [45] as the reference, resulting in 110 independent genetic variants that we used as IVs. The two IV sets provided a partial confirmation of associations due to having different strengths; while IV set A was derived from a subsample of UK Biobank that did not overlap the sample used to estimate SNP-outcome effects, it is more likely to be underpowered, whereas IV set B maximizes power at the cost of introducing possible bias due to sample overlap. Recent simulations have indicated that bias due to sample overlap is minimal when using Mendelian randomization and is often negligible compared to other biases, such as winner’s curse and weak-instrument bias [46, 47]. We estimated potential bias due to sample overlap using the method proposed by Burgess et al [47], implemented in an online tool (https://sb452.shinyapps.io/overlap/). The same SNP-outcome effects were used for MR of both IV sets.

We estimated the SNP-outcome effects (SNP-TRD or SNP-AD) for the SNPs in each IV set in the UK Biobank using logistic regression, adjusting for the first six genetic PCs. Four complementary MR methods were used to estimate the effect of vitamin D on each depression subtype: the random-effects IVW, the weighted median estimator, the weighted mode estimator, and MR-Egger regression. These methods have different strengths and assumptions; they take into account heterogeneity between SNPs (random-effects IVW), are unbiased if up to 50% of the information from the IVs is invalid (weighted median) [48], are unbiased if a weighted plurality of the genetic variants are valid (weighted mode) [49], are unbiased even if all IVs are invalid (MR-Egger regression) under a weaker set of assumptions [50].

We calculated the proportion of variance explained (PVE) in vitamin D for each SNP used as an IV for vitamin D using the effects and standard errors from the summary results, for both IV sets. We also performed a post hoc power calculation for the MR analyses using the method proposed by Brion et al. [51], implemented in an online tool (https://shiny.cnsgenomics.com/mRnd/).

## Results

### Observational analysis

Sample characteristics for each depression subtype in the observational analyses are provided in Table 1. We observed differences between TRD (*n* = 830 incident cases) and non-TRD subsets for almost all characteristics examined, except for the season of blood draw where only minimal differences were evident. Similar differences were observed for atypical depression (*n* = 2366 cases) and non-atypical depression subsets.

**Table 1:** Sample characteristics in incident treatment-resistant (TRD) cases and atypical depression (AD) cases compared to controls in observational analyses.

	TRD			AD		
	Cases	Controls		Cases	Controls	
	*(n* = *830)*	*GP controls (n* = *203,112)*	*MDD controls (n* = *16,694)*	*(n* = *2366)*	*MHQ controls (n* = *138,016)*	*MDD controls (n* = *28,232)*
	*Mean* ± *SD, N (%)*	*Mean* ± *SD, N (%)*	*Mean* ± *SD, N (%)*	*Mean* ± *SD, N (%)*	*Mean* ± *SD, N (%)*	*Mean* ± *SD, N (%)*
Age (years)	54.83 ± 8.13	56.45 ± 8.10	55.41 ± 8.02	51.70 ± 7.22	55.92 ± 7.75	54.05 ± 7.55
Sex						
*Female*	592 (71.33)	109257 (53.79)	11298 (67.68)	1740 (73.54)	76727 (55.59)	19651 (69.61)
*Male*	238 (28.67)	93855 (46.21)	5396 (32.32)	626 (26.46)	61289 (44.41)	8581 (30.39)
Ethnicity						
*Non-white*	44 (5.3)	8863 (4.36)	587 (3.52)	104 (4.4)	3846 (2.79)	744 (2.64)
*White*	786 (94.7)	194249 (95.64)	16107 (96.48)	2262 (95.6)	134170 (97.21)	27488 (97.36)
TDI*	−0.56 ± 3.37	−1.36 ± 3.02	−1.05 ± 3.11	−0.83 ± 3.24	−1.73 ± 2.82	−1.51 ± 2.92
BMI	29.31 ± 5.99	27.5 ± 4.81	28.20 ± 5.38	30.60 ± 5.81	26.69 ± 4.51	26.88 ± 4.90
Alcohol consumption						
*Daily*	104 (12.53)	40016 (19.7)	2780 (16.65)	348 (14.71)	32212 (23.34)	5597 (19.83)
*1–4 times weekly*	325 (39.16)	101065 (49.76)	7480 (44.81)	953 (40.28)	70859 (51.34)	14201 (50.3)
*1–3 times monthly*	287 (34.58)	45917 (22.61)	4781 (28.64)	833 (35.21)	27456 (19.89)	6578 (23.3)
*Never*	114 (13.73)	16114 (7.93)	1653 (9.9)	232 (9.81)	7489 (5.43)	1856 (6.57)
Smoking						
*Current*	125 (15.06)	21146 (10.41)	2429 (14.55)	258 (10.9)	9794 (7.1)	2662 (9.43)
*Previous*	288 (34.7)	70428 (34.67)	5896 (35.32)	890 (37.62)	48658 (35.26)	9975 (35.33)
*Never*	417 (50.24)	111538 (54.91)	8369 (50.13)	1218 (51.48)	79564 (57.65)	15595 (55.24)
Season of blood draw						
*Spring*	248 (29.88)	59713 (29.4)	5105 (30.58)	717 (30.3)	39004 (28.26)	8020 (28.41)
*Summer*	203 (24.46)	49786 (24.51)	4098 (24.55)	636 (26.88)	36939 (26.76)	7535 (26.69)
*Autumn*	205 (24.7)	49028 (24.14)	3944 (23.63)	549 (23.2)	34080 (24.69)	7092 (25.12)
*Winter*	174 (20.96)	44585 (21.95)	3547 (21.25)	464 (19.61)	27993 (20.28)	5585 (19.78)

Note: For TRD, control groups were either whole sample controls or probable MDD cases (that did not meet criteria for TRD) defined from primary care (GP) data. For AD, controls were either whole sample controls from participants responding to the MHQ or probable MDD cases that did not meet criteria for AD defined from self-reported symptoms in the Mental Health Questionnaire (MHQ). *TDI—Townsend deprivation index

There was no evidence that levels of serum vitamin D were associated with risk of TRD when compared to probable MDD cases, used as controls (adjusted model OR per SD increase in vitamin D 1.02 [95% CI 0.95, 1.10]), shown in Table 2. There was some evidence of an association between higher vitamin D levels and decreased risk of AD when compared to probable MDD cases used as controls (adjusted model OR 0.93 [95% CI 0.88, 0.97]).

**Table 2:** Observational associations of serum vitamin D with depression subtypes (TRD—treatment-resistant depression; AD—atypical depression) when using probable MDD cases as controls and secondly when using whole sample controls.

		Probable MDD controls			Whole sample controls		
Model	Outcome	OR [95% CI]	*P*-value	*N*	OR [95% CI]	*P*-value	*N*
*Base*	TRD	0.949 [0.886,1.02]	0.140178	17524	0.901 [0.842,0.965]	0.002816	203942
*Adjusted*	TRD	1.02 [0.947,1.1]	0.567396	17524	1.01 [0.937,1.09]	0.781148	203942
*Base*	AD	0.783 [0.749,0.817]	1.87E−28	30598	0.769 [0.738,0.802]	2.62E−34	140382
*Adjusted*	AD	0.927 [0.882,0.974]	0.002718	30598	0.917 [0.874,0.962]	0.000362	140382

Effects are presented as odds ratios (OR) per standard deviation (SD) increase in vitamin D. The base model includes adjustment for age and sex. The fully adjusted models include the adjustment for BMI, TDI, ethnicity, smoking, alcohol consumption frequency, and season of blood draw.

When comparing cases to whole-sample (general population) controls, there was evidence of an association between vitamin D and incident TRD in the base model (adjusted for age and sex), as shown in Table 2, with a one SD increase in vitamin D associated with a decreased risk of TRD (OR 0.90 [95% CI 0.84, 0.97]). Evidence of an association attenuated once adjusted for additional covariates (OR 1.01 [95% CI 0.94, 1.09]). Similarly, for AD, there was evidence that vitamin D decreased risk of AD in the base model (OR 0.77 [95% CI 0.74, 0.80]), shown in Table 2, which attenuated but remained significant after adjustment for additional covariates (adjusted OR 0.92 [95% CI 0.87, 0.96]). In a sensitivity analysis using whole sample controls with probable MDD cases excluded we observed highly concordant results with the main analysis for both TRD and AD (Supplementary Table 1).

### Genetic analysis

Sample characteristics for the subset of UK Biobank with genetic data for each depression subtype are provided in Supplementary Table 2.

Vitamin D PRS strongly associated with serum vitamin D, with the most predictive PRS threshold for the vitamin D calculated using PRSice being at a *p*-value threshold of 10^−5^ (Supplementary Fig. 1). The *R*^2^ of the association between vitamin D PRS and serum vitamin D at the most predictive *p*-value threshold was 0.026, with 21 SNPs included. Adjustment for the season of blood draw did not affect the results (Supplementary Fig. 2). There was no evidence of an association between vitamin D PRS and prevalent TRD (OR per SD increase in PRS 0.998 [95%CI 0.95,1.05], *n* = 1891 cases, *n* = 176693 controls) or AD (OR per SD increase in PRS 0.997 [95%CI 0.96, 1.04], *n* = 2101 cases, *n* = 124025 controls). In a sensitivity analysis, further adjustment for an assessment centre, genotype batch in UK Biobank, had little effect on the results (Supplementary Table 3). In a further sensitivity analysis using whole sample controls with probable MDD cases excluded, we found little difference in estimated effects (Supplementary Table 4).

In a power calculation for the PRS analyses, we estimated that we had 85% power to detect an association between vitamin D PRS and TRD and 94% power to detect an association for AD, assuming a genetic correlation of 0.2.

### Mendelian randomization

In the two-sample MR, for IV set A, we used 27 independent SNPs that were genome-wide significant in the vitamin D GWAS performed using the subsample of UK Biobank without MHQ or primary care data, explaining 3.6% of the variance in vitamin D. Of the 27 genome-wide significant independent SNPs used as IVs in IV set A, 25 were reported as genome-wide significant in the whole-sample GWAS of vitamin D in UK Biobank [19], with the remaining two being in LD with at least one variant reported as genome-wide significant. For IV set B, 110 SNPs were used as IVs, explaining ~19% of the variance in vitamin D, after clumping of the summary results of the GWAS of vitamin D in the whole-sample UK Biobank (Supplementary Tables 5, 6).

**Fig. 1:**
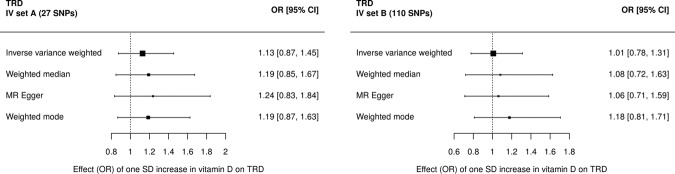
Two-sample Mendelian randomization (MR) of vitamin D treatment-resistant depression (TRD). MR using (**a**) the 27 IVs from IV set A, from the GWAS of vitamin D in the UK Biobank split sample, or (**b**) the 110 IVs from IV set B, from the previous GWAS of vitamin D.

**Fig. 2:**
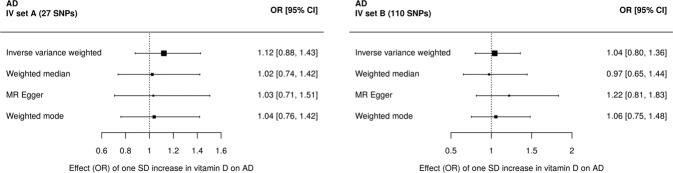
Two-sample Mendelian randomization (MR) of vitamin D and a typical depression (AD). MR using (**a**) the 27 IVs from IV set A, from the GWAS of vitamin D in the UK Biobank split sample or (**b**) the 110 IVs from IV set B, from the previous GWAS of vitamin D.

Two-sample Mendelian randomization analyses provided little evidence of an effect of vitamin D on prevalent TRD when using IV set A (27 SNPs, IVW OR 1.13 [95% CI 0.87, 1.45]) or IV set B (110 SNPs, IVW OR 1.01 [95%CI 0.78, 1.31]) (Fig. 1 and Supplementary Tables 7, 8). Effects differed by MR estimation method used with wide confidence intervals that overlapped the null for all methods. There was no evidence of an association between vitamin D and prevalent AD when using IV set A (27 SNPs, IVW OR 1.12 [95%CI 0.88, 1.43]) or IV set B (110 SNPs, IVW OR 1.04 [95%CI 0.80, 1.36]) in the two-sample Mendelian randomization (Fig. 2 and Supplementary Tables 9, 10). The direction of effects differed by MR estimation method used with wide confidence intervals that overlapped the null for all methods.

A leave-one-out-analysis did not suggest that there was any substantial effect of outlier SNPs for either of the two sets of IVs (Supplementary Figs. 3–6). We found no evidence for heterogeneity amongst IVs in either IV set when using the IVW or MR Egger regression methods and no evidence for directional horizontal pleiotropy in the MR Egger regression (Supplementary Tables 11, 12). We tested for a difference between estimates from the observational analyses and the MR but found limited evidence of heterogeneity between the two (Supplementary Results section A). A sensitivity analysis using whole sample controls with the exclusion for probable MDD cases applied showed similar results to the main analyses, for both TRD and AD, with little evidence of an association (Supplementary Tables 13, 14).

For TRD, assuming a sample size of 178,584 (proportion of cases = 0.011) and a minimum variance explained in vitamin D by the genetic variants of 3.6%, we had 80% power to detect an OR of 1.33 and 100% power to detect an OR larger than 1.54 per SD increase in vitamin D, at an alpha of 0.05. For AD, assuming a sample size of 126,126 (proportion of cases = 0.017) and the same variance explained by vitamin D SNPs, we had 80% power to detect an OR of 1.32 and 100% power to detect an OR larger than 1.53 per SD increase in vitamin D, at an alpha of 0.05.

We also estimated the potential bias incurred by using overlapping samples in IV set B (Supplementary Table 15). For an exposure sample size of 417,580 and a conservative variance explained of 3.6% there was a maximum bias of 0.004 (logOR) per SD increase in vitamin D and a type 1 error rate that remained at 0.05, at a 100% sample overlap for AD. For TRD, there was similarly a maximum bias of 0.004 (logOR) per SD increase in vitamin D and type 1 error rate that remained at 0.05, at a sample overlap of 100%.

## Discussion

The comprehensive genetic analyses that included PRS and two-sample MR performed in the current study did not provide evidence for a causal link between genetically determined vitamin D levels and either TRD or AD. These findings are in keeping with previous genetic analyses which have also reported no causal associations between vitamin D levels and MDD [19, 21–24]. Results do not support a causal role for vitamin D in the two MDD subtypes investigated and indicate that caution be exercised when considering further trialling of vitamin D in these patient groups. To date, trials of vitamin D supplementation for MDD have largely produced mixed findings [10–12, 52], with no specific data pertaining to MDD subtypes. A recent trial sought to determine whether administering vitamin D as an adjunct to antidepressants might improve depression severity in those with TRD [53]. However, futility had to be declared based largely on an inability to enrol suitable participants. The current genetic analysis raises a question about whether further attempts should be made to trial vitamin D in this patient subtype.

Previous epidemiological studies have reported longitudinal associations between serum vitamin D levels and depression [9, 54–56]. The fact that genetic evidence contradicts these findings suggests that residual confounding and reverse causation should be considered as alternative explanations for the role of vitamin D in depression and its subtypes. In a recent paper where we reported prospective associations between vitamin D levels and depressive symptoms in a large sample of UK Biobank participants [9], we posited that vitamin D might be a manifestation of separate underlying predisposing factors for depression. There is evidence that decreasing vitamin D levels might be a biomarker of poor physical health [57] which may play a role in the development of depression (Ronaldson et al. [9]) and may therefore have predictive utility. Lower levels of vitamin D might also be a consequence of developing or subthreshold depression in that depressive symptoms might cause a reduction in physical activity and sunlight exposure leading to vitamin D deficiency (i.e., reverse causation). Previous MR studies have shown an effect of MDD on vitamin D levels [19, 24], suggesting that reverse causation might also be present in MDD subtypes (TRD and AD). However, we could not investigate the bidirectional relationship between depression subtypes and vitamin D in the current study due to the low number of cases (and hence low statistical power) to identify genome-wide significant genetic variants associated with each MDD subtype.

### Strengths and limitations

The current study has several strengths. The large population size and the broad range of measures in UK Biobank allowed for comprehensive adjustment of relevant covariates in the observational analyses. Data integration in the UK Biobank from multiple sources (primary care data and MHQ) allowed us to investigate novel, potentially clinically relevant MDD subtypes that had not, to the best of our knowledge, been examined before in relation to vitamin D. The use of multiple complementary analytical approaches (observational and genetic data; PRS and MR) in an attempt at evidence triangulation, strengthens our conclusions. We a priori viewed the MR analyses as complementary to the observational analyses, in pursuit of evidence triangulation, rather than an attempt at confirmation of any associations found in the observational analyses. The two approaches should be seen as complementary because they have different strengths and limitations; in the observational analyses, we restricted to cases of TRD or AD that occurred after vitamin D measurement, whereas in MR we can examine risk across the lifetime and hence use prevalent cases. Lastly, by using two-sample MR we were able to utilize summary results from published GWAS of vitamin D in large samples as opposed to relying solely on individual-level data.

Several limitations need consideration. Firstly, we were unable to adjust observational associations for certain factors known to affect vitamin D levels, such as anti-epileptic drugs and corticosteroids [58]. However, we expect MR associations to be less subject to biases induced by these factors. Secondly, we cannot rule out the possibility that small effects of vitamin D on depression subtypes exist that we were unable to detect due to limited statistical power. Similarly, the effects of vitamin D on other depression subtypes may exist but have not been investigated. Thirdly, MR assumes a linear relationship between exposure and outcome that is constant throughout the life-course (i.e., from the point in the life-course where the genetic variant has been associated with the exposure). Very low concentrations (marked deficiency) of vitamin D may be associated with the depression subtypes investigated, or vitamin D levels in childhood may predispose an individual to later life TRD or AD. However, given that observational studies detect an association between vitamin D and depression at these periods in the life-course, we consider these possibilities unlikely. Fourthly, estimates of statistical power in the MR may be inflated due to overfitting and winner’s curse since the variance explained by the vitamin D SNPs was calculated from the same population used for the discovery analysis (GWAS) and may therefore be higher than if calculated in an external sample. Fifthly, the previous whole-sample GWAS of vitamin D from which we derived IVs for IV set B adjusted for additional covariates (sex, age, assessment centre, genotype batch, month of measurement) that we did not adjust for in the GWAS of vitamin D used to derive IV set A. Our MR results, however, appear to be robust to these methodological differences, with consistent results across the two IV sets indicating little evidence of an association for either MDD subtype. Finally, results may not be fully generalizable to the UK population since the UK Biobank is known to not be fully representative of the UK population [59]. Likewise, our results are likely not generalizable to non-European ancestries as analyses were conducted using data from samples of broadly European ancestry. Since vitamin D deficiency is known to be more prevalent in people of non-European ancestry [60], effects may differ in other samples.

## Conclusion

Our comprehensive investigation of the effects of vitamin D on two potentially clinically relevant subtypes of depression; TRD and AD, indicated some evidence of a weak association observationally, but little evidence of association when using genetic evidence from PRS and Mendelian randomization. These results mirror similar conclusions of genetic studies of vitamin D on broad MDD phenotypes. Although our results do not support further clinical trials in these depression subtypes, they do not rule out that small effects may exist that require larger sample sizes to detect.

## Supplementary information

Supplementary Material
