# Supplementary material for: Vitamin D and the risk of treatment-resistant and atypical depression: A Mendelian randomization study
Source: Transl Psychiatry. 2021 Nov 4;11:561. doi: 10.1038/s41398-021-01674-3 (PMC8568901; doi:10.1038/s41398-021-01674-3)
Supplement: Supplementary file 1 — Supplementary Material [file 41398_2021_1674_MOESM1_ESM.docx]

**Supplementary Material**

**Supplementary Methods**

**Genetic data QC**

Using genetic principal components (PCs) provided by the UK Biobank, 4-means clustering on the first two PCs was performed to identify and select individuals of European ancestry. QC was then performed using PLINK v1.9(1) to remove: variants with missingness > 0.02 (before individual QC), individuals with missingness > 0.02, individuals whose self-reported sex was discordant from their genetic sex, variants with missingness > 0.02 (after individual QC), variants departing from Hardy-Weinberg Equilibrium (p < 10e-8), and variants with minor allele frequency (MAF) < 0.01. Relatedness kinship estimates provided by the UK Biobank were used to identify pairs of related individuals using KING(2) (r2 > 0.044) and the GreedyRelated algorithm was used to remove one individual from each pair, preferentially retaining individuals that survived previous QC. FlashPCA(3) was used to generate PCs for the sub-set of individuals of European ancestry that remained following exclusions.

**GWAS of vitamin D**

For the mixed model GWAS using regenie(4) we retained related individuals but excluded individuals of non-European ancestry. We used rank inverse-normal transformed vitamin D levels. Variants with imputation quality (INFO) scores <0.9 and minor allele frequency (MAF) <0.01 were excluded. We selected independent genome-wide significant variants for both IV sets by clumping summary results at an r2 of 0.001 and 10000kb window, using 1000 Genomes as the references panel.

**Supplementary Results section A**

We tested for an interaction between the significant observational association for AD (adjusted for covariates) and the estimate from the two-sample MR using IV set B for AD, using the formula described by Altman et al(5). There was little evidence to support a difference between the two estimates (z=-0.92, p>0.05), however inference the test was limited due to sample overlap between the observational and MR analyses.

**References**

1. Purcell S, Neale B, Todd-Brown K, Thomas L, Ferreira MAR, Bender D, et al. PLINK: A Tool Set for Whole-Genome Association and Population-Based Linkage Analyses. The American Journal of Human Genetics. 2007 Sep 1;81(3):559–75.

2. Manichaikul A, Mychaleckyj JC, Rich SS, Daly K, Sale M, Chen W-M. Robust relationship inference in genome-wide association studies. Bioinformatics. 2010 Nov 15 [cited 2021 Aug 17];26(22):2867–73. Available from: https://academic.oup.com/bioinformatics/article/26/22/2867/228512

3. Abraham G, Inouye M. Fast Principal Component Analysis of Large-Scale Genome-Wide Data. PLOS ONE . 2014 Apr 9 [cited 2021 Aug 17];9(4):e93766. Available from: https://journals.plos.org/plosone/article?id=10.1371/journal.pone.0093766

4. Mbatchou J, Barnard L, Backman J, Marcketta A, Kosmicki JA, Ziyatdinov A, et al. Computationally efficient whole-genome regression for quantitative and binary traits. Nature Genetics 2021 53:7 . 2021 May 20 [cited 2021 Aug 17];53(7):1097–103. Available from: https://www.nature.com/articles/s41588-021-00870-7

5. Altman DG, Bland JM. Interaction revisited: the difference between two estimates. BMJ. 2003 Jan 25 [cited 2021 Aug 17];326(7382):219. Available from: https://www.bmj.com/content/326/7382/219


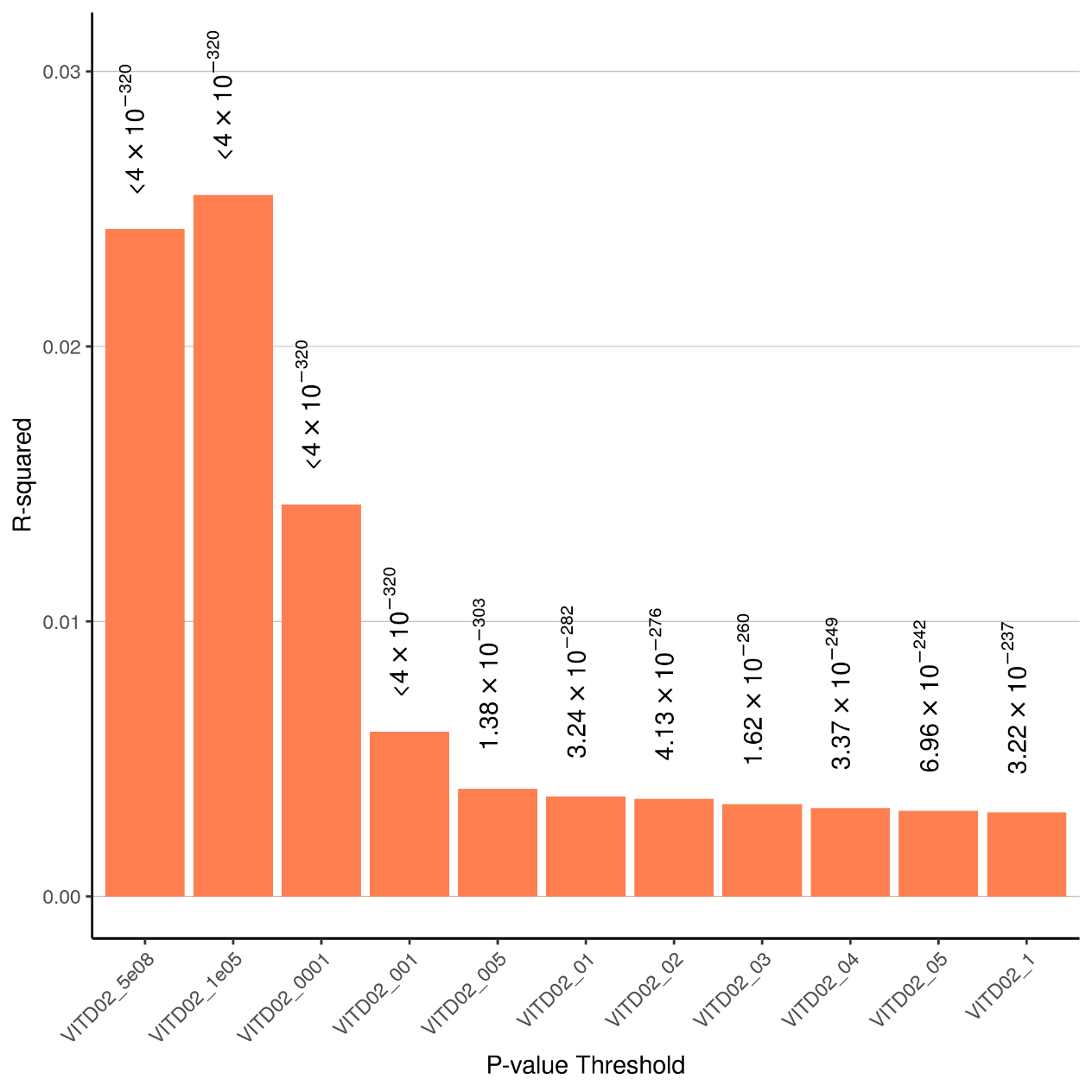


Figure 1 Validation of vitamin D PRS at 11 p-value thresholds in UK Biobank. Note that p-values below 4x10^-320^ are labelled as “<4x10^-320^”.


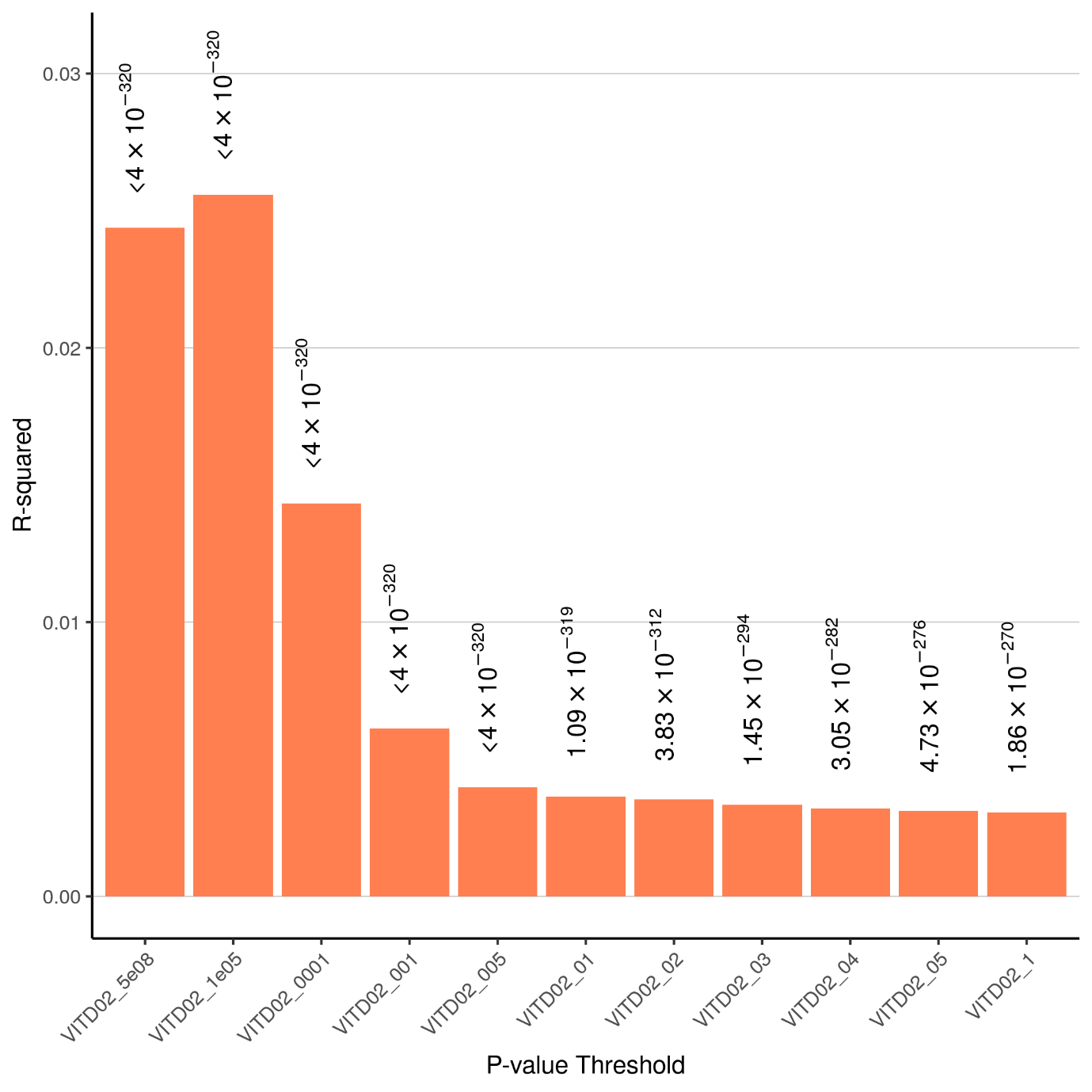


Figure 2 Sensitivity analysis of the validation of vitamin D PRS at 11 p-value thresholds in UK Biobank, adjusting additionally for season of blood draw. Note that p-values below 4x10-320 are labelled as “<4x10-320”.

Table 1 Sensitivity analysis using whole sample controls with probable MDD cases excluded in the observational analysis.

| **Model** | **Outcome** | **OR [95% CI]** | **P-value** | **N** |
| --- | --- | --- | --- | --- |
| *Base* | *TRD* | *0.897[0.838,0.961]* | *0.00184* | *187248* |
| *Adjusted* | *TRD* | *1.01[0.936,1.090]* | *0.80488* | *187248* |
| *Base* | *AD* | *0.767[0.735,0.800]* | *8.59E-35* | *112150* |
| *Adjusted* | *AD* | *0.918[0.875,0.963]* | *0.000509* | *112150* |

Table 2 Sample characteristics in prevalent treatment resistant depression (TRD) cases and atypical depression (AD) cases compared to controls in the subset of UK Biobank with genetic data, used for PRS analyses and for estimating SNP-outcome effects in Mendelian randomization.

|  | **TRD** |  | **AD** |  |
| --- | --- | --- | --- | --- |
|  | **Cases** | **Controls** | **Cases** | **Controls** |
| **N** | *1891* | *176693* | *2101* | *124025* |
| **Sex (female)** | *1367(72.3)* | *95592(54.1)* | *1553(73.9)* | *69359(55.9)* |
| **Age** | *56(7.8)* | *57(8)* | *52(7.1)* | *56(7.7)* |

Table 3 Sensitivity analysis of vitamin D PRS on prevalent TRD and AD, adjusting additionally for assessment centre and genotype batch.

| **Outcome** | **Effect (OR) [95%CI]** | **SE** | **P-value** |
| --- | --- | --- | --- |
| *TRD* | *0.998[0.954,1.045]* | *0.02315* | *0.943* |
| *AD* | *0.997[0.956,1.042]* | *0.02199* | *0.913* |

Table 4 Sensitivity analysis of vitamin D PRS on prevalent TRD and AD using whole sample controls screened for MDD.

| **Outcome** | **Effect (OR) [95%CI]** | **SE** | **P-value** |
| --- | --- | --- | --- |
| *TRD* | *0.998[0.954,1.045]* | *0.0232* | *0.942* |
| *AD* | *0.994[0.952,1.038]* | *0.0221* | *0.799* |

Table 5 Summary results of previously published GWAS of vitamin D in UK Biobank by Revez et al. genome-wide significant independent SNPs used as IVs in two-sample MR.

| **Chrom** | **SNP** | **Effect allele** | **EAF** | **Beta** | **SE** | **P-value** | **PVE*** |
| --- | --- | --- | --- | --- | --- | --- | --- |
| 1 | rs6671730 | G | 0.566 | 0.015 | 0.002 | 1.92E-13 | 0.000681 |
| 1 | rs35408430 | C | 0.658 | 0.021 | 0.002 | 1.36E-24 | 0.001319 |
| 1 | rs7522116 | C | 0.434 | 0.013 | 0.002 | 2.97E-11 | 0.000557 |
| 1 | rs2131925 | G | 0.356 | 0.023 | 0.002 | 3.61E-28 | 0.001524 |
| 1 | rs7528419 | A | 0.775 | -0.020 | 0.002 | 1.35E-16 | 0.000861 |
| 1 | rs12123821 | C | 0.952 | -0.079 | 0.005 | 2.55E-63 | 0.003542 |
| 1 | rs61815559 | A | 0.969 | -0.086 | 0.006 | 4.27E-51 | 0.00284 |
| 1 | rs10908469 | A | 0.730 | -0.016 | 0.002 | 2.49E-13 | 0.000675 |
| 1 | rs6672758 | C | 0.199 | -0.018 | 0.003 | 2.40E-12 | 0.000619 |
| 2 | rs6547409 | C | 0.950 | -0.026 | 0.005 | 1.30E-08 | 0.000407 |
| 2 | rs1260326 | T | 0.393 | -0.021 | 0.002 | 4.42E-24 | 0.001289 |
| 2 | rs727857 | G | 0.389 | 0.014 | 0.002 | 1.05E-11 | 0.000582 |
| 2 | rs58235267 | C | 0.512 | 0.012 | 0.002 | 8.79E-09 | 0.000417 |
| 2 | rs3849374 | G | 0.822 | 0.016 | 0.003 | 7.46E-10 | 0.000477 |
| 2 | rs7569755 | G | 0.709 | -0.014 | 0.002 | 1.18E-10 | 0.000523 |
| 2 | rs1047891 | C | 0.684 | 0.015 | 0.002 | 1.18E-12 | 0.000636 |
| 2 | rs2012736 | C | 0.919 | 0.048 | 0.004 | 1.16E-39 | 0.002184 |
| 3 | rs6550617 | A | 0.282 | 0.012 | 0.002 | 2.19E-08 | 0.000394 |
| 3 | rs11721204 | C | 0.562 | -0.014 | 0.002 | 1.17E-11 | 0.000579 |
| 3 | rs6782190 | G | 0.352 | 0.017 | 0.002 | 1.45E-16 | 0.000859 |
| 3 | rs6438900 | C | 0.742 | -0.014 | 0.002 | 3.07E-09 | 0.000443 |
| 3 | rs9861009 | T | 0.272 | -0.014 | 0.002 | 4.86E-10 | 0.000488 |
| 4 | rs78649910 | T | 0.894 | 0.021 | 0.003 | 7.15E-11 | 0.000535 |
| 4 | rs4364259 | G | 0.798 | -0.016 | 0.003 | 2.16E-10 | 0.000508 |
| 4 | rs4616820 | C | 0.535 | 0.012 | 0.002 | 1.13E-09 | 0.000467 |
| 4 | rs7439366 | T | 0.544 | 0.032 | 0.002 | 1.41E-58 | 0.00327 |
| 4 | rs7675923 | A | 0.156 | -0.026 | 0.003 | 1.63E-20 | 0.001085 |
| 4 | rs4694423 | C | 0.584 | 0.101 | 0.002 | 9.995e-313 | 0.030325 |
| 4 | rs71601787 | G | 0.673 | -0.042 | 0.002 | 1.22E-86 | 0.00488 |
| 4 | rs7657132 | A | 0.683 | 0.014 | 0.002 | 5.14E-11 | 0.000543 |
| 4 | rs55814693 | G | 0.700 | 0.013 | 0.002 | 2.45E-09 | 0.000448 |
| 4 | rs11732896 | G | 0.701 | 0.016 | 0.002 | 1.79E-13 | 0.000683 |
| 4 | rs189407772 | A | 0.977 | -0.053 | 0.007 | 3.90E-15 | 0.000777 |
| 5 | rs10070734 | T | 0.290 | -0.013 | 0.002 | 1.82E-09 | 0.000455 |
| 5 | rs31612 | T | 0.826 | 0.015 | 0.003 | 4.15E-08 | 0.000379 |
| 5 | rs9325107 | G | 0.558 | -0.011 | 0.002 | 3.23E-08 | 0.000385 |
| 6 | rs72834856 | T | 0.928 | 0.025 | 0.004 | 8.67E-11 | 0.00053 |
| 6 | rs28374650 | C | 0.756 | 0.014 | 0.002 | 5.44E-09 | 0.000429 |
| 6 | rs9476310 | C | 0.489 | -0.012 | 0.002 | 4.21E-09 | 0.000435 |
| 6 | rs9490317 | T | 0.554 | -0.011 | 0.002 | 3.95E-08 | 0.00038 |
| 6 | rs2248551 | G | 0.835 | 0.023 | 0.003 | 3.04E-18 | 0.000955 |
| 7 | rs10085881 | T | 0.718 | 0.015 | 0.002 | 7.83E-11 | 0.000533 |
| 7 | rs7784802 | A | 0.639 | -0.014 | 0.002 | 2.62E-11 | 0.00056 |
| 7 | rs75741381 | C | 0.852 | 0.017 | 0.003 | 4.15E-09 | 0.000435 |
| 7 | rs6966728 | C | 0.537 | 0.012 | 0.002 | 8.01E-09 | 0.000419 |
| 7 | rs2346264 | A | 0.217 | 0.014 | 0.002 | 1.20E-08 | 0.000409 |
| 8 | rs804281 | A | 0.416 | -0.013 | 0.002 | 4.72E-11 | 0.000545 |
| 8 | rs28692966 | G | 0.747 | -0.015 | 0.002 | 1.13E-10 | 0.000524 |
| 8 | rs57459725 | C | 0.867 | 0.018 | 0.003 | 2.12E-09 | 0.000452 |
| 8 | rs12056768 | T | 0.417 | 0.023 | 0.002 | 6.44E-31 | 0.001681 |
| 9 | rs13284054 | T | 0.882 | -0.018 | 0.003 | 2.07E-08 | 0.000396 |
| 9 | rs9409266 | G | 0.139 | 0.020 | 0.003 | 9.45E-12 | 0.000585 |
| 9 | rs532436 | G | 0.816 | 0.019 | 0.003 | 5.31E-13 | 0.000656 |
| 10 | rs77532868 | C | 0.946 | -0.027 | 0.004 | 1.56E-09 | 0.000459 |
| 10 | rs3925446 | G | 0.801 | -0.015 | 0.002 | 1.09E-09 | 0.000468 |
| 11 | rs117862422 | T | 0.986 | 0.055 | 0.009 | 2.32E-10 | 0.000506 |
| 11 | rs143488652 | A | 0.987 | 0.064 | 0.009 | 1.91E-13 | 0.000681 |
| 11 | rs138072379 | C | 0.981 | -0.054 | 0.007 | 1.33E-13 | 0.00069 |
| 11 | rs182244780 | G | 0.987 | 0.357 | 0.009 | 9.995e-313 | 0.020449 |
| 11 | rs10832277 | G | 0.631 | 0.074 | 0.002 | 9.995e-313 | 0.015829 |
| 11 | rs146128209 | A | 0.929 | 0.058 | 0.004 | 1.86E-49 | 0.002746 |
| 11 | rs33981819 | T | 0.539 | -0.012 | 0.002 | 1.05E-09 | 0.000469 |
| 11 | rs1660870 | A | 0.667 | -0.019 | 0.002 | 1.19E-18 | 0.000978 |
| 11 | rs12803256 | A | 0.223 | -0.104 | 0.002 | 9.995e-313 | 0.023219 |
| 11 | rs609799 | C | 0.821 | -0.015 | 0.003 | 1.19E-08 | 0.000409 |
| 11 | rs964184 | G | 0.132 | -0.043 | 0.003 | 1.09E-48 | 0.002702 |
| 11 | rs2847500 | G | 0.876 | 0.022 | 0.003 | 4.42E-13 | 0.00066 |
| 12 | rs12317268 | A | 0.849 | 0.021 | 0.003 | 6.19E-14 | 0.000709 |
| 12 | rs11182428 | T | 0.480 | 0.013 | 0.002 | 3.23E-10 | 0.000498 |
| 12 | rs1038165 | C | 0.417 | -0.012 | 0.002 | 2.31E-09 | 0.00045 |
| 12 | rs10859995 | T | 0.417 | 0.040 | 0.002 | 1.06E-88 | 0.004999 |
| 12 | rs12372115 | G | 0.929 | 0.022 | 0.004 | 1.93E-08 | 0.000398 |
| 12 | rs73413596 | T | 0.926 | -0.022 | 0.004 | 1.41E-08 | 0.000405 |
| 13 | rs9569209 | C | 0.714 | 0.013 | 0.002 | 9.35E-09 | 0.000415 |
| 14 | rs10146891 | C | 0.644 | -0.013 | 0.002 | 8.04E-10 | 0.000475 |
| 14 | rs8018720 | G | 0.177 | 0.038 | 0.003 | 1.26E-47 | 0.002641 |
| 14 | rs4906378 | C | 0.661 | 0.013 | 0.002 | 1.19E-09 | 0.000466 |
| 15 | rs1532085 | A | 0.385 | -0.026 | 0.002 | 1.55E-37 | 0.002061 |
| 15 | rs1800588 | C | 0.785 | 0.033 | 0.002 | 4.38E-42 | 0.002323 |
| 15 | rs62012766 | T | 0.843 | 0.017 | 0.003 | 3.53E-10 | 0.000496 |
| 15 | rs62007299 | G | 0.287 | 0.013 | 0.002 | 1.32E-09 | 0.000463 |
| 15 | rs325384 | C | 0.716 | 0.014 | 0.002 | 1.66E-10 | 0.000514 |
| 16 | rs10083762 | C | 0.727 | -0.014 | 0.002 | 1.48E-09 | 0.00046 |
| 16 | rs77924615 | G | 0.807 | 0.017 | 0.003 | 7.11E-11 | 0.000535 |
| 16 | rs8063565 | G | 0.266 | -0.015 | 0.002 | 5.57E-11 | 0.000541 |
| 16 | rs11076175 | A | 0.822 | -0.023 | 0.003 | 9.48E-19 | 0.000984 |
| 16 | rs139861017 | C | 0.983 | -0.043 | 0.008 | 3.04E-08 | 0.000386 |
| 16 | rs4327060 | C | 0.946 | 0.024 | 0.004 | 2.92E-08 | 0.000387 |
| 16 | rs11542462 | G | 0.866 | 0.023 | 0.003 | 1.27E-15 | 0.000805 |
| 17 | rs10454087 | C | 0.715 | 0.014 | 0.002 | 8.70E-10 | 0.000473 |
| 17 | rs2952289 | C | 0.202 | -0.018 | 0.002 | 1.18E-12 | 0.000636 |
| 18 | rs8091117 | C | 0.935 | 0.026 | 0.004 | 5.98E-11 | 0.000539 |
| 18 | rs4121823 | T | 0.155 | 0.019 | 0.003 | 3.83E-12 | 0.000607 |
| 18 | rs656384 | G | 0.734 | 0.013 | 0.002 | 1.31E-08 | 0.000407 |
| 18 | rs2037511 | G | 0.834 | -0.018 | 0.003 | 1.35E-11 | 0.000576 |
| 19 | rs142158911 | G | 0.885 | -0.026 | 0.003 | 4.78E-16 | 0.000829 |
| 19 | rs12462826 | G | 0.631 | 0.012 | 0.002 | 2.70E-09 | 0.000446 |
| 19 | rs8107974 | A | 0.924 | -0.040 | 0.004 | 6.49E-26 | 0.001394 |
| 19 | rs3814995 | C | 0.688 | 0.013 | 0.002 | 5.18E-09 | 0.00043 |
| 19 | rs12721051 | C | 0.814 | 0.016 | 0.003 | 1.81E-10 | 0.000512 |
| 19 | rs212100 | T | 0.164 | 0.066 | 0.003 | 9.995e-313 | 0.007561 |
| 19 | rs1048328 | G | 0.920 | -0.028 | 0.004 | 8.66E-15 | 0.000758 |
| 20 | rs2207132 | G | 0.967 | 0.035 | 0.006 | 5.56E-10 | 0.000484 |
| 20 | rs6123359 | A | 0.898 | -0.034 | 0.003 | 6.10E-25 | 0.001339 |
| 20 | rs2585442 | C | 0.759 | -0.036 | 0.002 | 6.70E-51 | 0.002829 |
| 20 | rs2616279 | C | 0.848 | 0.023 | 0.003 | 3.89E-16 | 0.000835 |
| 21 | rs2229742 | G | 0.897 | 0.025 | 0.003 | 1.48E-14 | 0.000744 |
| 22 | rs6003465 | T | 0.668 | 0.012 | 0.002 | 1.77E-08 | 0.0004 |
| 22 | rs2074735 | G | 0.936 | -0.028 | 0.004 | 8.23E-12 | 0.000588 |
| 22 | rs115621755 | C | 0.673 | 0.012 | 0.002 | 4.76E-09 | 0.000432 |

*PVE – proportion variance explained

Table 6 Summary results of the genome-wide significant independent variants from the GWAS of vitamin D in the subsample of UK Biobank with no MHQ or linked primary care data, used as IVs in the two-sample MR.

| **Chrom** | **SNP** | **Effect allele** | **EAF** | **Beta** | **SE** | **P-value** | **PVE*** |
| --- | --- | --- | --- | --- | --- | --- | --- |
| 1 | rs61815559 | A | 0.966307 | -0.09332 | 0.009855 | 2.81E-21 | 0.000599 |
| 1 | rs12122629 | A | 0.957234 | -0.0714 | 0.008706 | 2.37E-16 | 0.000449 |
| 1 | rs3750296 | G | 0.657052 | 0.027114 | 0.00366 | 1.28E-13 | 0.000367 |
| 1 | rs12239736 | T | 0.65749 | -0.02638 | 0.003709 | 1.13E-12 | 0.000338 |
| 2 | rs12475068 | C | 0.91492 | 0.051594 | 0.00626 | 1.70E-16 | 0.000454 |
| 2 | rs1260326 | T | 0.3955 | -0.01989 | 0.003557 | 2.23E-08 | 0.000209 |
| 3 | rs9861009 | T | 0.272752 | -0.02232 | 0.003923 | 1.27E-08 | 0.000216 |
| 4 | rs222059 | A | 0.438138 | -0.13408 | 0.003556 | 1.00E-300 | 0.009412 |
| 4 | rs28577591 | G | 0.688801 | -0.04788 | 0.003787 | 1.18E-36 | 0.001068 |
| 4 | rs11934762 | T | 0.519047 | 0.03165 | 0.0035 | 1.52E-19 | 0.000546 |
| 6 | rs2608953 | C | 0.834562 | 0.028142 | 0.004683 | 1.86E-09 | 0.000241 |
| 8 | rs7828742 | A | 0.400587 | 0.022089 | 0.003568 | 5.98E-10 | 0.000256 |
| 10 | rs12242871 | G | 0.471131 | 0.020723 | 0.003485 | 2.73E-09 | 0.000236 |
| 11 | rs117913124 | G | 0.972858 | 0.377512 | 0.010838 | 7.62E-266 | 0.008045 |
| 11 | rs12793530 | G | 0.215681 | -0.10444 | 0.004265 | 1.91E-132 | 0.003993 |
| 11 | rs7128011 | G | 0.630179 | 0.076918 | 0.00364 | 4.00E-99 | 0.002976 |
| 11 | rs964184 | G | 0.13244 | -0.04801 | 0.005156 | 1.26E-20 | 0.000579 |
| 11 | rs113140528 | A | 0.815447 | -0.03364 | 0.004572 | 1.87E-13 | 0.000362 |
| 11 | rs36037728 | C | 0.973992 | 0.062596 | 0.011011 | 1.31E-08 | 0.000216 |
| 11 | rs117832548 | A | 0.985209 | 0.084096 | 0.015025 | 2.18E-08 | 0.000209 |
| 12 | rs10859995 | T | 0.417581 | 0.044872 | 0.003535 | 6.48E-37 | 0.001076 |
| 14 | rs2144530 | C | 0.176612 | 0.0436 | 0.004551 | 9.76E-22 | 0.000613 |
| 15 | rs7170361 | T | 0.71564 | 0.033207 | 0.003944 | 3.78E-17 | 0.000474 |
| 15 | rs174418 | T | 0.402407 | -0.02428 | 0.003551 | 8.12E-12 | 0.000312 |
| 16 | rs12720922 | G | 0.816913 | -0.02694 | 0.004506 | 2.23E-09 | 0.000239 |
| 19 | rs112285002 | C | 0.836445 | -0.07197 | 0.004711 | 1.09E-52 | 0.001558 |
| 20 | rs6127099 | A | 0.720552 | 0.035238 | 0.003959 | 5.51E-19 | 0.000529 |

*PVE – proportion variance explained

Table 7 Two-sample MR results for vitamin D on TRD using IV set A and four complementary estimation methods.

| **Outcome** | **Exposure** | **Method** | **SNPs** | **Beta*** | **SE** | **P-value** |
| --- | --- | --- | --- | --- | --- | --- |
| TRD | Vitamin D | Weighted median | 27 | 0.175 | 0.174 | 0.313 |
| TRD | Vitamin D | Inverse variance weighted | 27 | 0.120 | 0.130 | 0.355 |
| TRD | Vitamin D | Weighted mode | 27 | 0.171 | 0.160 | 0.295 |
| TRD | Vitamin D | MR Egger | 27 | 0.213 | 0.202 | 0.302 |

*Effects are presented as logOR for TRD per SD increase in vitamin D.

Table 8 Two-sample MR results for vitamin D on TRD using IV set B and four complementary estimation methods

| **Outcome** | **Exposure** | **Method** | **SNPs** | **Beta*** | **SE** | **P-value** |
| --- | --- | --- | --- | --- | --- | --- |
| TRD | Vitamin D | Weighted median | 110 | 0.079 | 0.208 | 0.704 |
| TRD | Vitamin D | Inverse variance weighted | 110 | 0.008 | 0.134 | 0.950 |
| TRD | Vitamin D | Weighted mode | 110 | 0.162 | 0.190 | 0.395 |
| TRD | Vitamin D | MR Egger | 110 | 0.060 | 0.204 | 0.768 |

* Effects are presented as logOR for TRD per SD increase in vitamin D

Table 9 Two-sample MR results for vitamin D on atypical depression (AD) using IV set A and four complementary estimation methods.

| **Outcome** | **Exposure** | **Method** | **SNPs** | **Beta*** | **SE** | **P-value** |
| --- | --- | --- | --- | --- | --- | --- |
| AD | Vitamin D | Weighted median | 27 | 0.023 | 0.168 | 0.891 |
| AD | Vitamin D | Inverse variance weighted | 27 | 0.114 | 0.124 | 0.356 |
| AD | Vitamin D | Weighted mode | 27 | 0.038 | 0.159 | 0.812 |
| AD | Vitamin D | MR Egger | 27 | 0.032 | 0.193 | 0.872 |

*Effects are presented as logOR for AD per SD increase in vitamin D.

Table 10 Two-sample MR results for vitamin D on AD using IV set B and four complementary estimation methods

| **Outcome** | **Exposure** | **Method** | **SNPs** | **Beta*** | **SE** | **P-value** |
| --- | --- | --- | --- | --- | --- | --- |
| *AD* | *Vitamin D* | *Weighted median* | *110* | *-0.029* | *0.202* | *0.887* |
| *AD* | *Vitamin D* | *Inverse variance weighted* | *110* | *0.041* | *0.136* | *0.760* |
| *AD* | *Vitamin D* | *Weighted mode* | *110* | *0.055* | *0.173* | *0.753* |
| *AD* | *Vitamin D* | *MR Egger* | *110* | *0.200* | *0.207* | *0.336* |

**Effects are presented as logOR for AD per SD increase in vitamin D.*

Table 11 Results of tests for directional horizontal pleiotropy using the MR Egger regression intercept term for IV sets A and B in the two-sample of MR of vitamin D on atypical depression (AD) and treatment-resistant depression (TRD).

| **Outcome** | **Exposure** | **Egger intercept** | **SE** | **P-value** | **IV set** |
| --- | --- | --- | --- | --- | --- |
| *AD* | *Vitamin D* | 0.006492 | 0.011653 | 0.582404 | A |
| *AD* | *Vitamin D* | -0.0061 | 0.006018 | 0.313216 | B |
| *TRD* | *Vitamin D* | -0.00732 | 0.01221 | 0.554119 | A |
| *TRD* | *Vitamin D* | -0.002 | 0.005937 | 0.737198 | B |

Table 12 Results of tests of heterogeneity for IV sets A and B in the two-sample of MR of vitamin D on atypical depression (AD) and treatment-resistant depression (TRD).

| **Outcome** | **Exposure** | **Method** | **Q** | **Q df** | **Q p-value** | **IV set** |
| --- | --- | --- | --- | --- | --- | --- |
| AD | Vitamin D | MR Egger | 19.18169 | 25 | 0.788327 | A |
| AD | Vitamin D | Inverse variance weighted | 19.49206 | 26 | 0.814996 | A |
| AD | Vitamin D | MR Egger | 121.6554 | 108 | 0.174237 | B |
| AD | Vitamin D | Inverse variance weighted | 122.8119 | 109 | 0.172752 | B |
| TRD | Vitamin D | MR Egger | 23.26188 | 25 | 0.562274 | A |
| TRD | Vitamin D | Inverse variance weighted | 23.62149 | 26 | 0.597599 | A |
| TRD | Vitamin D | MR Egger | 99.79686 | 108 | 0.700997 | B |
| TRD | Vitamin D | Inverse variance weighted | 99.91004 | 109 | 0.721785 | B |

Table 13 Sensitivity analysis using whole samples controls with probable MDD cases excluded in the two-sample MR for TRD.

| **IV set** | **Outcome** | **Exposure** | **Method** | **SNPs** | **Beta*** | **SE** | **P-value** |
| --- | --- | --- | --- | --- | --- | --- | --- |
| A | TRD | Vitamin D | Weighted median | 27 | 0.16022 | 0.182916 | 0.381071 |
| A | TRD | Vitamin D | Inverse variance weighted | 27 | 0.118474 | 0.129783 | 0.361314 |
| A | TRD | Vitamin D | Weighted mode | 27 | 0.181482 | 0.157401 | 0.259405 |
| A | TRD | Vitamin D | MR Egger | 27 | 0.210739 | 0.202007 | 0.306825 |
| B | TRD | Vitamin D | Weighted median | 110 | -0.03446 | 0.203447 | 0.865496 |
| B | TRD | Vitamin D | Inverse variance weighted | 110 | 0.023885 | 0.13555 | 0.860129 |
| B | TRD | Vitamin D | Weighted mode | 110 | 0.053353 | 0.174328 | 0.760151 |
| B | TRD | Vitamin D | MR Egger | 110 | 0.172946 | 0.20712 | 0.405558 |

Table 14 Sensitivity analysis using whole samples controls with probable MDD cases excluded in the two-sample MR for AD.

| **IV set** | **Outcome** | **Exposure** | **Method** | **SNPs** | **Beta*** | **SE** | **P-value** |
| --- | --- | --- | --- | --- | --- | --- | --- |
| A | AD | Vitamin D | Weighted median | 27 | 0.017497 | 0.162866 | 0.914447 |
| A | AD | Vitamin D | Inverse variance weighted | 27 | 0.099968 | 0.123869 | 0.419641 |
| A | AD | Vitamin D | Weighted mode | 27 | 0.00927 | 0.15706 | 0.953385 |
| A | AD | Vitamin D | MR Egger | 27 | 0.016058 | 0.193276 | 0.934448 |
| B | AD | Vitamin D | Weighted median | 110 | -0.03446 | 0.203447 | 0.865496 |
| B | AD | Vitamin D | Inverse variance weighted | 110 | 0.023885 | 0.13555 | 0.860129 |
| B | AD | Vitamin D | Weighted mode | 110 | 0.053353 | 0.174328 | 0.760151 |
| B | AD | Vitamin D | MR Egger | 110 | 0.172946 | 0.20712 | 0.405558 |

Table 15 Estimated bias incurred due to sample overlap in the two-sample Mendelian randomization analyses using IV set B, for different levels of bias to the observational (ordinary least squares – OLS) estimate.

| **Outcome** | **Bias of the OLS estimate*** | **Overlap proportion** | **Bias incurred*** | **Type 1 error rate** |
| --- | --- | --- | --- | --- |
| AD | 0.1 | 1 | 0.001 | 0.05 |
| AD | 0.2 | 1 | 0.002 | 0.05 |
| AD | 0.3 | 1 | 0.002 | 0.05 |
| AD | 0.4 | 1 | 0.003 | 0.05 |
| AD | 0.5 | 1 | 0.004 | 0.05 |
| TRD | 0.1 | 1 | 0.001 | 0.05 |
| TRD | 0.2 | 1 | 0.002 | 0.05 |
| TRD | 0.3 | 1 | 0.002 | 0.05 |
| TRD | 0.4 | 1 | 0.003 | 0.05 |
| TRD | 0.5 | 1 | 0.004 | 0.05 |

**Bias of observational and IV estimates are log odds ratios for the outcome per standard deviation change in the risk factor.*


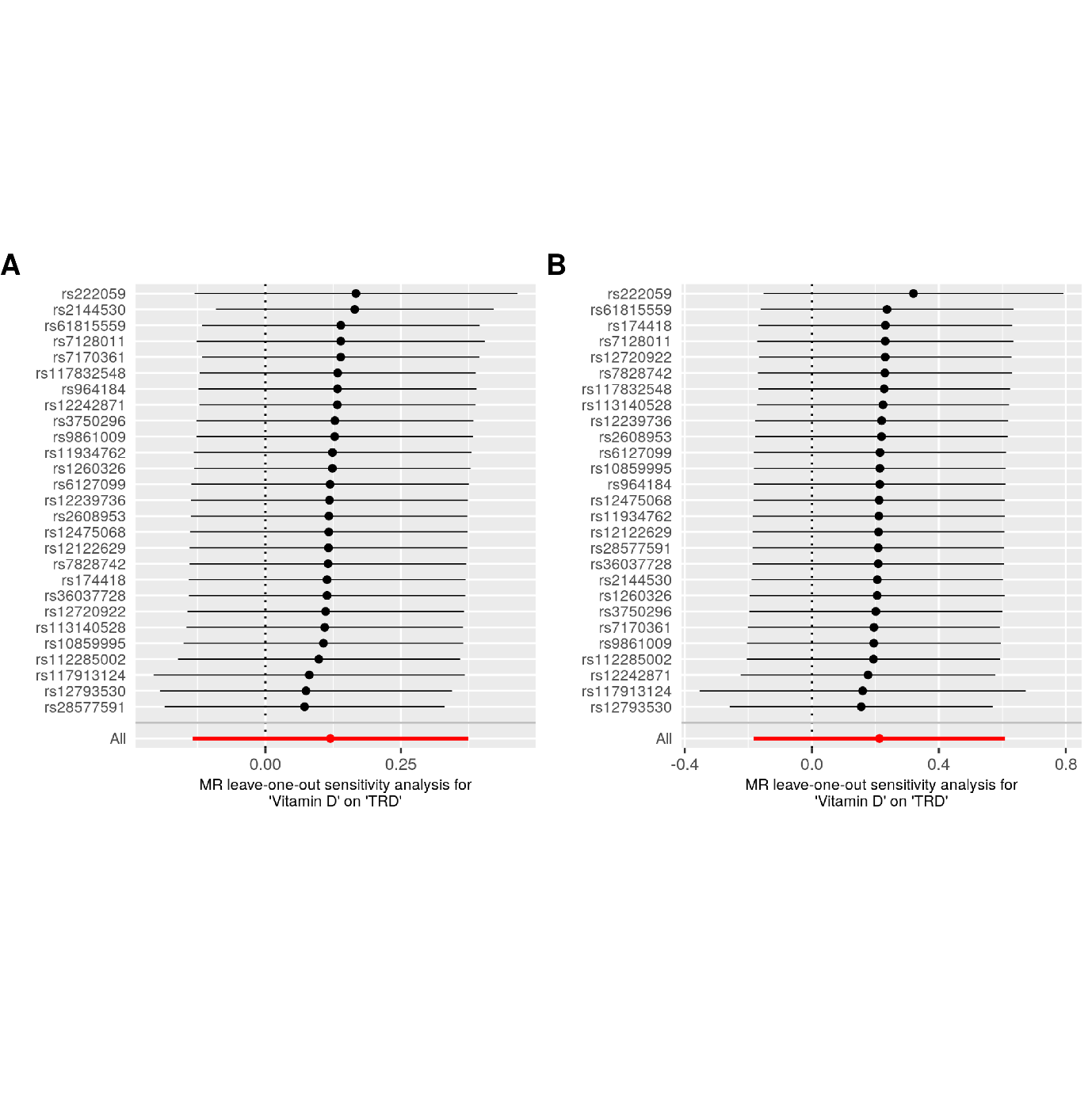


Figure 3 Leave-one-out analysis of MR of vitamin D on TRD using IV set A for A) the IVW and B) MR Egger regression estimates.


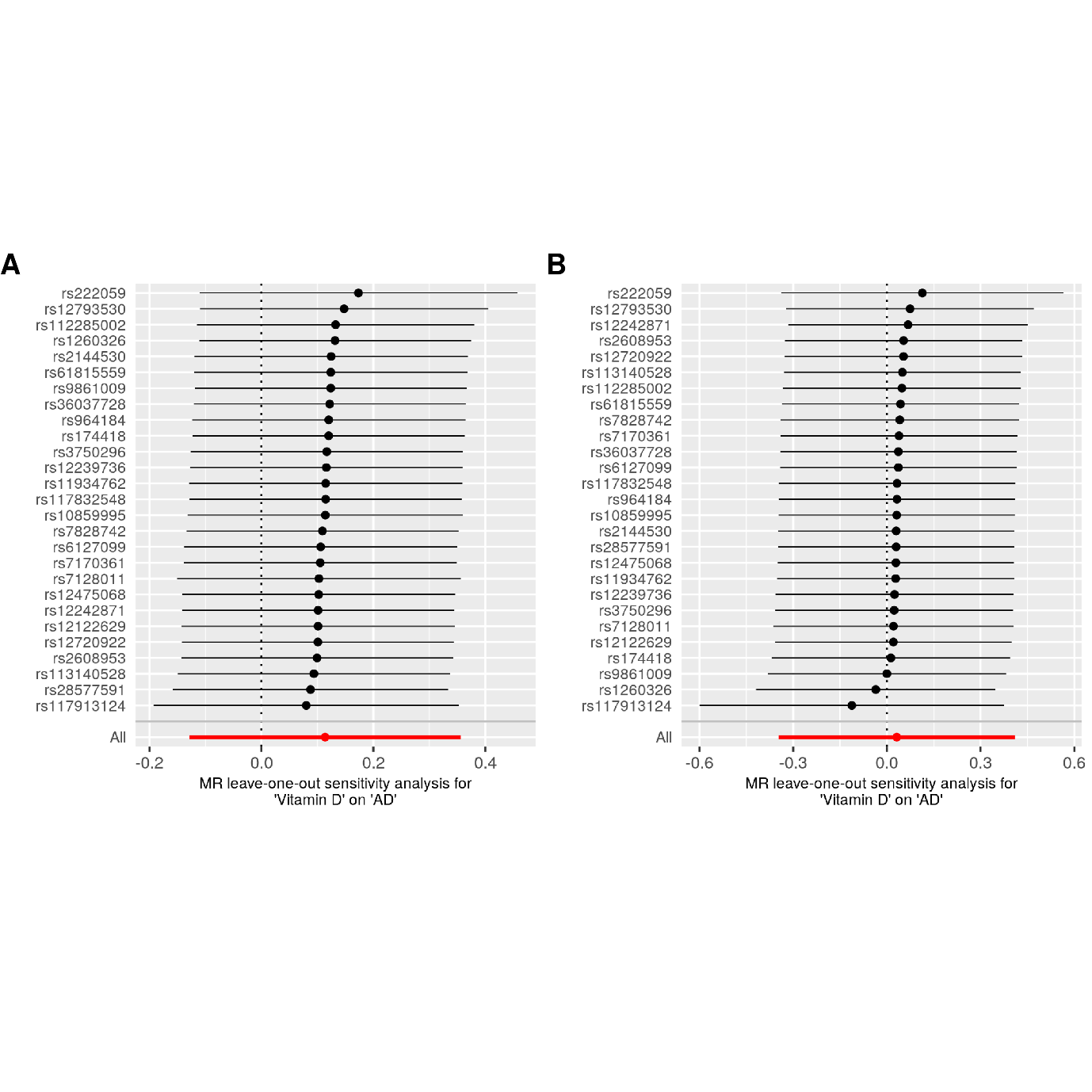


Figure 4 Leave-one-out analysis of MR of vitamin D on AD using IV set A for A) the IVW and B) MR Egger regression estimates.


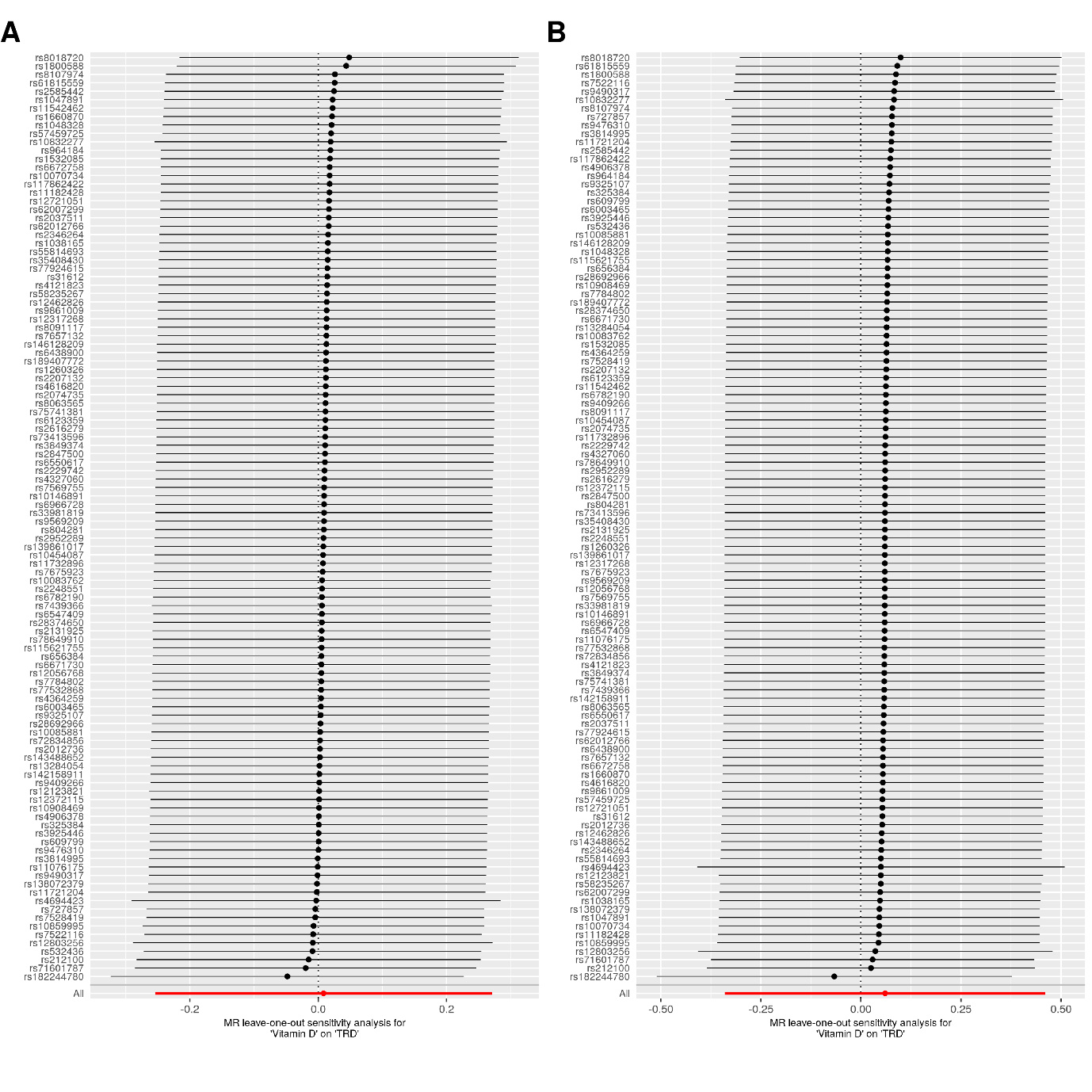


Figure 5 Leave-one-out analysis of MR of vitamin D on TRD using IV set B for A) the IVW and B) MR Egger regression estimates.


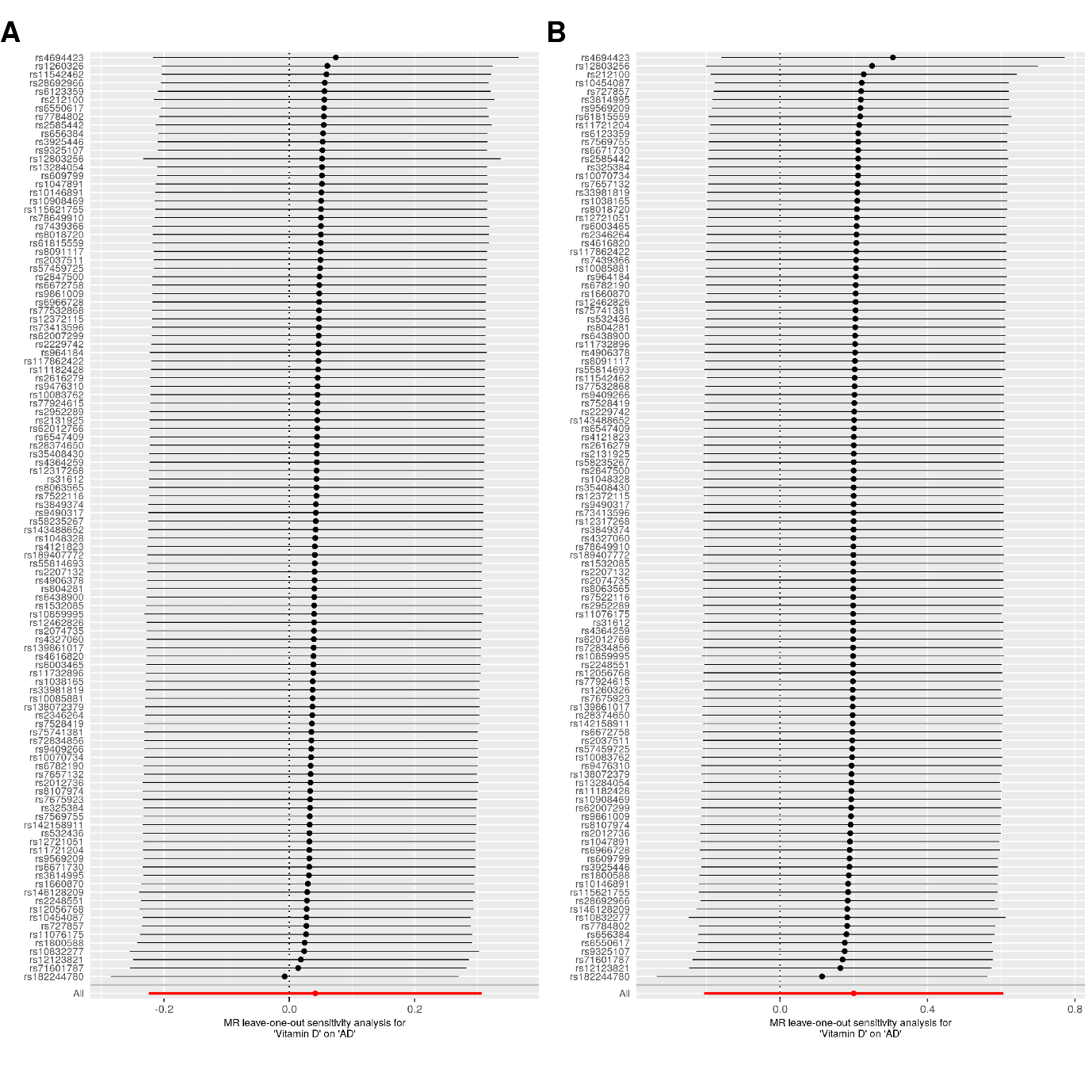


Figure 6 Leave-one-out analysis of MR of vitamin D on AD using IV set B for A) the IVW and B) MR Egger regression estimates.
